# Conflict, community, and collaboration: shared implementation barriers and strategies in two polio endemic countries

**DOI:** 10.1186/s12889-020-09235-x

**Published:** 2020-12-18

**Authors:** Eme Owoaje, Ahmad Omid Rahimi, Anna Kalbarczyk, Oluwaseun Akinyemi, Michael A. Peters, Olakunle O. Alonge

**Affiliations:** 1 University of Ibadan College of Medicine, Ibadan, Nigeria; 2 Global Innovations Consultancy Services, Kabul, Afghanistan; 3 Department of International Health, Johns Hopkins Bloomberg School of Public Health, 615 N Wolfe St., Baltimore, MD USA

**Keywords:** Polio, Endemic, Conflict, Vaccine hesitancy, Mistrust, Community engagement

## Abstract

**Background:**

Afghanistan and Nigeria are two of the three remaining polio endemic countries. While these two countries have unique sociocultural characteristics, they share major polio risk factors. This paper describes the countries’ shared contexts and highlights important lessons on implementing polio eradication activities among hard-to-reach populations relevant for future global health programs.

**Methods:**

A grey literature review of the Global Polio Eradication Initiative (GPEI) followed by an online survey was conducted in both countries. The survey was targeted to individuals who have been involved continuously in polio eradication activities for 12 months or more since 1988. A sub-set of respondents from the survey was recruited for key-informant interviews (KII). The survey and KIIs were conducted between September 2018–April 2019. A cross-case comparison analysis was conducted to describe shared implementation challenges, strategies, and unintended consequences of polio eradication activities across these contexts.

**Results:**

Five hundred thirteen and nine hundred twenty-one surveys were completed in Afghanistan and Nigeria respectively; 28 KIIs were conducted in Afghanistan and 29 in Nigeria. Major polio eradication activities in both countries include house-to-house campaigns, cross-border stations, outreach to mobile populations, and surveillance. Common barriers to these activities in both countries include civil unrest and conflict; competing political agendas; and vaccine refusal, fatigue, and mistrust, all of which are all bases for describing hard-to-reach populations. Both countries employed strategies to engage community leadership, political and religious groups through advocacy visits, and recruited community members to participate in program activities to address misconceptions and distrust. Recruitment of female workers has been necessary for accessing women and children in conservative communities. Synergy with other health programs has been valuable; health workers have improved knowledge of the communities they serve which is applicable to other initiatives.

**Conclusions:**

The power of community engagement at all levels (from leadership to membership) cannot be overstated, particularly in countries facing civil unrest and insecurity. Workforce motivation, community fatigue and mistrust, political priorities, and conflict are intricately interrelated. Community needs should be holistically assessed and addressed;programs must invest in the needs of health workers who engage in these long-term health programs, particularly in unsafe areas, to alleviate demotivation and fatigue.

## Background

Afghanistan and Nigeria are two of the three remaining polio endemic countries globally; neither country has been able to successfully interrupt all types of polio, with transmission of wild poliovirus (WPV) ongoingin Afghanistan and circulating vaccine-derived poliovirus (cVDPV) occurring in Nigeria. Concerted efforts of the global community, national governments, and local actors have resulted in great strides towards polio eradication in both countries, yet there is still work to be done. Given the fact that the two countries are budgeted to receive over US$900 million from the Global Polio Eradication Initiative (GPEI) between 2019 and 2023 (about 22% of the overall GPEI budget), a consideration of the lessons learned in implementation to date can provide timely insight to improve future activities in these two contexts [1]. Furthermore, knowledge gained through polio eradication efforts can be distilled and applied to improve the efficiency and efficacy of other global public health programs in the future. Despite numerous reports and recommendations from GPEI partners [2], the Independent Monitoring Board (IMB) [3], academic authors [4], and others, few attempts have been made to systematically study and compare the lessons learned by the vast range of stakeholders committed to eradicating polio in these contexts.

Research to map knowledge and identify lessons learned from polio eradication activities was conducted in Afghanistan and Nigeria under the Synthesis and Translation of Research and Innovations from Polio Eradication (STRIPE) consortium, a collaboration between Johns Hopkins University (JHU) and 7 academic and research institutions representing countries with different epidemiologic profiles of polio (described previously in this supplement) [5]. This cross-case comparison seeks to describe the preliminary findings for two polio-endemic countries, outlining broadly shared challenges and lessons learned in program implementation.. Though separated by continents, culture, and politics, these two countries share important risk factors for the spread of polio including civil unrest and conflict; vaccine refusals, mistrust, and fatigue; and competing political agendas. Many of these countries’ shared experiences are grounded in the aforementioned risk factors which continue to disrupt the polio program today. Understanding how these shared risk factors emerge across different contexts to affect polio eradication efforts will yield important lessons for improving the effectiveness of the polio program, and developing strategies for managing disease outbreaks and building resilient health systems.

## Methods

The STRIPE consortium employed an explanatory mixed method design [6], comprising of a grey literature review, a quantitative survey, and key informant interviews to describe lessons learned in various countries and globally.

A country-level grey literature review was included as one component of an overall effort to review literature generated through various polio eradication initiatives. The grey literature review aimed to trace national and sub-national experiences within the GPEI. We included materials that described aspects of GPEI implementation from January 01, 1988 through late 2018. Data were extracted across a range of implementation science components (e.g. strategies and outcomes) as well as other variables including global health knowledge areas, relevance to World Health Organization (WHO) health systems building blocks, and examples, development, or use of tools, manuals, and guidelines.

To identify participants for the quantitative and qualitative components, each country team developed a theoretical list of all actors directly involved in implementing polio eradication related activities for 12 or more continuous months between 1988 to date, otherwise known as the country’s polio universe. In short, a polio universe constitutes all polio-related actors in a study area [7].

The polio universe in Afghanistan was described using the WHO health system building blocks as a framework [8] and by using different levels of health service delivery and current national guidelines for polio eradication [9]. The building blocks framework presents the key elements of the health system including 1) service delivery, 2) workforce, 3) medical supplies, 4) governance, 5) health information, and 6) finance. The universe was first described using the six building blocks as sub-headings and potential research units were enumerated in relation to national and subnational levels of operation. The research units included Emergency Operation Centers (EOCs), Expanded Programme on Immunization (EPI) departments, WHO, United Nations Children’s Fund (UNICEF), and implementing non-government organizations (NGOs).

In Nigeria, the polio universe was comprised of individuals working with the government of Nigeria (at national, state, and local levels), multilateral agencies, as well as international and local non-governmental organisations involved in polio eradication activities for at least 12 months continuously between 1988 and today. To facilitate sampling from the universe in Nigeria, survey respondents were purposively selected from at least one state in each of the six geopolitical zones. The states selected in each zone were: South West (Ondo, Oyo, and Lagos), South-South (Bayelsa), South-East (Anambra), North West (Kano, Sokoto), North Central (Nasarawa), and North East (Borno). Detailed descriptions and rationales for the construction of country-level polio universe are described elsewhere [7].

### Data collection

A standard questionnaire was administered to individuals within respective polio universes to better understand the challenges faced in polio eradication and identify lessons learned in both countries. The questionnaire was designed using constructs from implementation science frameworks (e.g. the Consolidated Framework for Implementation Research (CFIR)) [10] to describe facilitators and barriers to the success of the polio programs, the strategies deployed to address barriers, and unintended consequences of polio-related activities. The CFIR presents domains of individual characteristics, organizational settings, GPEI program design, process of implementation, and external settings as factors that could have contributed to program success and failures. These domains are described in detail elsewhere [10] and are the basis for analysis of the quantitative portion of this research. The questionnaire was pretested in English at JHU by experts who had been involved in polio eradication research and/or policymaking and was then piloted by local researchers after translation into local language(s). Minor clarifying edits were incorporated and the final questionnaire was administered online and by trained interviewers in both countries.

Survey data were analyzed to ascertain each country’s largest challenges in polio eradication. Named change agents at national and field levels who responded to the survey, indicated that they played a role in working to resolve these challenges, and agreed to participate in follow-up activities were identified by each country’s study team and selected to participate in Key Informant Interviews (KIIs). Semi-structured KIIs were conducted by trained qualitative researchers on the country teams using a tool designed from the Socioecological Model (SEM) [11]. The SEM considers the complex relationships between factors that influence the individual, interpersonal, organization, community, and larger environment. The KII tool asked participants to describe challenges, solutions, and contextual factors at each of these levels. Transcripts were translated to English by country teams and centrally organized by the JHU team.

The survey and KII methodologies are described in further detail elsewhere [5]. The study protocols were reviewed and approved by each country’s Institutional Review Board. Surveys were conducted from October – November 2018 in Afghanistan followed by KII fieldwork from January – March 2019. In Nigeria, surveys were conducted from September 2018 - January 2019 followed by KIIs from January–April, 2019.

### Data analysis

Country-specific findings were intiailly synthesized by the country’s research team, followed by an inductive analysis to identify common themes across countries. Quantitative data was summarized to describe respondent characteristics and identify factors that were barriers and/or facilitators to specific polio program goals. Major shared factors were explained in greater detail using qualitative data from the KIIs. Mapping these barriers to levels of the SEM and using rich text from key informant interviews allowed for explanations into the processes by which they obstructed polio program success. An inductive content analysis approach was used to identify common themes for both countries across the SEM [12]. Country data was then organized along these themes to create a country case study.

## Results

Survey data mapped to each level in the SEM and illustrative quotations are displayed in Table 1. This model recognizes that individuals are embedded in larger organizational, social, political, and environmental systems and that actors and processes at one level influence and are influenced by actors and processes at the others [13]. Emergent themes across the different SEM levels included the role of conflict and insecurity (environment), competing political priorities (policy), and community engagement, vaccine hesitancy, mistrust and fatigue in different communities (social) many of which are deeply interrelated. Results from each country are reported separately below, organized by the four themes.

**Table 1:** Joint display of common barriers reported by each country in the survey and KIIs categorized by socioecological levels

Socioecological Level	Reported barrier at each SE level	Percent of responses by country	Illustrative quotes
**Environmental**	Overall^a^	AF 69.0% (*n* = 550/797) NG 38.3% (*n* = 984/2570)	*One thing is the territory controlled by Taliban that is 40–50% more or less. We have two problems there. Sometimes they ban polio program for example they banned the program in southern region, Kandahar, Helmand and Urozgan. There a million children were deprived of vaccines. This was a big challenge. Even if they allow the program they don’t allow house to house campaigns and instead they tell us to go site to site or mosque to mosque. In mosque to mosque, many children are missed especially the neonatal. People don’t bring the neonatal to the mosque. Second challenge is the campaign quality in these areas. Our monitor cannot go to Taliban controlled areas and cannot ensure reporting, so the campaign quality is compromised. - KII Afghanistan*
**Political/Social**	Social (subset of environmental level)	AF 42.8% of all environmental barriers reported (*n* = 262/612) NG 23.4% of all environmental barriers reported (*n* = 324/1379)	*They think it is made of haram material. They think the vaccine will convert our children non-obedient and impolite or they reach adulthood quickly. - KII Afghanistan*.
	Political (subset of environmental level)	AF 25.2% of all environmental barriers reported (*n* = 154/612) NG 22.0% of all environmental barriers reported (*n* = 3030/1379)	*There are times whereby there are clashes of activities, there may be a program polio eradication, side by side with another equally very important program, so in that case there are lots of clashes and you know it’s always not easy. I mean in some other cases, there are so many activities while the state is planning its own, maybe the ministry is calling you for one other activity, the National are planning their own. There was a scenario whereby we were having the last OBR that is Outbreak Response and National were coming with the CHIPS program, community health influencers promoter services which immunization is a component of it, you understand, so there is always this clash from below, within and above, so it’s not always easy. – KII Nigeria*
**Organizational**	Overall^a^	AF 9.8% (*n* = 39/797) NG 12.8% (*n* = 330/2570)	*… it [the polio program] raises expectations that the government should do more. Sometimes the polio program is taken as hostage. People do not allow vaccination because there is no drinking water or too much garbage in a village nobody plans to pick them up. They say why they polio vaccine while their basic needs are not addressed. - KII Afghanistan* *funds are always not sufficient to carry out planned activities, again equipment, infrastructure are not always sufficient. And I said again, the issue of acceptability s there and attitude of staff. – KII Nigeria*
	Individual perceptions of the organization (subset of individual level)	AF 28% of all individual barriers reported (*n* = 22/85) NG 17% of all individual barriers reported (*n* = 116/669)	*they cannot provide accurate and reliable information to the people. - KII Afghanistan*
**Individual**	Overall^a^	AF 8.5% (*n* = 68/797) NG 17.5% (*n* = 449/2570)	*Practitioners working in curative medicine in provinces also doubt the polio vaccines and argue how many doses should be given to children? In many occasion even those educated doctors have refused use vaccine. This refusal happened in accessible areas. This is creating suspicions in the community for other people. These practitioners should be educated on the benefit of vaccines and the side effects. – KII Afghanistan* *Burning out, yes, because it can be tough, it can be tough, especially during implementation you eat only once a day, and you are working under the sun throughout sometimes you meet armed robbers along the way, otherwise you are taking the challenges as they come. – KII Nigeria*

*Abbreviations*: *AF* Afghanistan, *NG* Nigeria, *SE* Socioecological

^a^Overall barriers are aligned with the first level of responses in the quantitative survey and can be compared directly across respondents within a certain country. Other aspects of the model were subsets of overall barriers and their denominators are not directly comparable as only respondents that met certain conditions were asked further questions

### Case study: Afghanistan

#### Respondent characteristics

Overall, 522 individuals attempted to complete the Afghanistan survey using the online and offline approaches combined, however 9 online responses were dropped from analysis due to incompleteness. The 513 remaining responses were obtained through a variety of methods; 365 face to face interviews were conducted in the selected provinces (Kabul: 113; Bamyan: 50; Herat: 42; Zabul: 30; Kandahar: 55; Helmand: 41; and Nangarhar: 50); 126 phone-surveys were completed in selected provinces (out of 442 phone numbers collected and contacted); and 22 participants completed online surveys. Twenty-eight KIIs were conducted in four of the aforementioned provinces based on availability, willingness to participate, level of involvement, and gender of previously identified respondents (Kabul: 11, Kandahar: 7, Bamyan: 5, and Nangarhar: 5). Table 2 displays the survey and KII respondents, their levels of involvement in the polio program, and organizational representation.

**Table 2:** Sample size and characteristics of respondents, Afghanistan and Nigeria

	Afghanistan		Nigeria	
	Survey (*n* = 513)	KIIs (*n* = 28)	Survey (*n* = 921)	KIIs (*n* = 29)
**Highest level of respondent’s involvement in polio eradication**				
National	**74**	**8**	**162**	**10**
Subnational	**386**	**13**	**662**	**13**
Frontline	**52**	**7**	**90**	**6**
*Missing*	**–**	**–**	**7**	**–**
**Organizational Affiliation(s)**				
GPEI partners^a^	**190**	**3**	**552**	**7**
Government	**173**	**16**	**671**	**2**
NGOs / implementing organizations	**243**	**8**	**248**	**20**
Research/academic orgs	**3**	**–**	**10**	**–**
Other	**–**	**1**	**–**	**–**

*Abbreviations*: *GPEI* Global Polio Eradication Initiative, *KIIs* Key Informant Interviews, *NGOs* Non-government Organizations

^a^GPEI partners include the World Health Organization (WHO), Rotarty International, the US Centers for Disease Control and Prevention (CDC), the United Nations Children’s Fund (UNICEF), and the Bill & Melinda Gates Foundation

Survey respondents identified barriers to program success for each polio-related activity in which they were engaged (e.g. vaccination, community engagement, and/or resource mobilization) (see Table 3). The most frequently reported barrierers were factors related to the external setting of the GPEI program, as these constituted 69.0% of total reported barriers in Afghanistan. The next most frequently reported barriers were factors related to the process of GPEI program implementation, followed by individual barriers, organizational barriers, and lastly, GPEI program characteristics.

**Table 3:** Types of barriers identified in GPEI program implementation in Afghanistan and Nigeria

Type of barriers	Number (percent) of total barriers identified	
	Afghanistan (*n* = 513 respondents)	Nigeria (*n* = 921 respondents)
External Setting	550 (.690)	984 (.383)
Process of Implementation	101 (.127)	516 (.201)
Individual	68 (.085)	449 (.175)
Organizational	39 (.049)	330 (.128)
GPEI Program Design	39 (.049)	291 (.113)
Total Barriers Identified	797	2570

*Abbreviations*: *GPEI* Global Polio Eradication Initiative

Individual = Characteristics of individuals of those associated with the organization involved in polio eradication activities

Organizational = Organizational settings and factors related to your organization supporting the polio eradication program

GPEI Program Design = Polio eradication program characteristics and the activity (ies) used towards eradication polio

Process of Implementation = Process of conducting the activities ie.g. how the activity was implemented, including the planning, execution strategies, reflection and evaluation of activities or adjustmenets made ot the plan

External setting = political, economic, social, technological or environmental settings in which the program operated

The majority of all respondents reported experiencing external barriers while attempting to eradicate polio in Afghanistan. 80% of respondents (*n* = 417) reported external barriers to program success. Political and social environmental factors constituted over 60% of all reported external barriers (42.8 and 25.2% respectively). External context emerged as the most significant challenge for those involved in community engagement (67% of all barriers cited).

#### Conflict and insecurity

Eradication efforts in Afghanistan have been complicated by civil unrest and insurgent occupation which makes some areas inaccessible to health workers who fear for their safety and security. One KII respondent at the subnational level said,

*“We are not going where we do not feel safe means we are not going to the insecure area. We do not go to such areas to put ourselves in danger but there is always a little risk, we monitor the situation or we are told by the office to avoid dangerous and insecure areas.”* - KII Afghanistan - Subnational level

The Afghanistan National Emergency Action Plan 2019 noted that even with complete implementation of planned activities, achieving success was contingent on accessibility which remains beyond program control [14]. The Taliban, a fundamentalist Islamic militia inAfghanistan and Pakistan, have imposed a ban on polio campaigns and actively target health workers with threats of kidnapping and death. As a result, vaccinators have been frequent targets of directed violence. One KII respondent in Afghanistan noted:

*One thing is the territory controlled by Taliban … We have two problems there. Sometimes they ban polio program for example they banned the program in southern region, Kandahar, Helmand and Urozgan. There, a million children were deprived of vaccines. This was a big challenge. Even if they allow the program they don’t allow house to house campaigns and instead they tell us to go site to site or mosque to mosque. In mosque to mosque, many children are missed especially the neonatal. People don’t bring the neonates to the mosque. Second challenge is the campaign quality in these areas. Our monitor cannot go to Taliban controlled areas and cannot ensure reporting, so the campaign quality is compromised*. - KII Afghanistan, National level

Given the negative influence of conflict and insecurity on polio program activities, one solution included coordination with different opposition groups to gain access to unsafe areas. In Afghanistan these were called ‘Days of Tranquility’ where all parties negotiate a cease-fire in order to allow children to access healthcare; these have contributed to higher vaccination coverage.

#### Competing political priorities

Political leaders in these conflict-affected areas have faced competing political priorities, balancing politically savvy messaging and other health priorities for the country. While government remains supportive of the polio program, high-profile endorsements could be counter-productive in insecure areas where anti-government forces may oppose government messaging. An extraction from the grey literature further describe this challenge:

*“In the case of Afghanistan, according to Toole et al (2009), while President Karzai wanted to bring an end to the Afghan war via a political settlement with the Taliban, they would not negotiate while US and foreign troops were in the country.”* [15]*. - Grey literature*.

The grey literature indicates that balancing political messaging for polio, together with conflict and insecurity continue to pose significant challenges to eradication activities.

#### Community engagement

Continuous engagement with communities in conflict areas has been an important strategy for the polio program. This is reflected by the fact that survey respondents involved in various program goals saw the social environment as the greatest external facilitator to program success (identified by 61.2% of all respondents).

Communities in Afghanistan were mobilized by engaging both religious and community leaders. Social mobilizers enlisted local influencers to support vaccine campaigns. Advocacy visits to these leaders helped improve communication and assuage religious and social concerns. One key-informant described this process:

*“Prior to every campaign, we meet the governor and other sectors department, like the HAJ and AWQAF – the department of religious affairs-- to send a written brief to every Masjid, and inform the locals about the dates of campaigns. Imams announce in Friday prayers that from this day vaccine campaign will start. So mullahs have to inform and announce it to people, and tell them to keep their children in houses for vaccine and explain that it is not forbidden in Islam.”* - KII Afghanistan, Subnational level

Community members were also incorporated into pre-implementation planning and vaccination activities. Preliminary, secondary, and high-school students were engaged in Afghanistan to serve as community mobilizers who increased awareness of the polio vaccine to their immediate family members and friends.

KII respondents also noted the importance of knowing the community well to reach every child. This included not only having the trust of the community and familiarity with community leaders but also with the settlement, the streets, and the households. For example, there are some communities in which only female health workers are allowed to enter the houses. Two frontline health workers reflected on the value of becoming familiar with communities and how it helped them achieve their goals:

*“If families did not get to know me during the awareness sessions, this would not have been possible. We go to these families to educate and to give them messages …*. *So we should know families at any case …*” - KII Afghanistan, Frontline worker

*“As the communication program started and social mobilizer teams are there in the field, now these social mobilizer teams are going to each house, meet them, so this way, they get familiar, they win peoples trust and the benefit of this program is, the people don’t prohibit vaccination anymore; so the vaccination program performs properly.”-* KII Afghanistan, Frontline worker

#### Mistrust, Hesitancy, and Fatigue

Even though community engagement has been integral to polio eradication activities, social barriers, especially mistrust and rejection of the program, emerged as the largest external barrier for survey respondents in Afghanistan. There has been substantial mistrust and rejection of the program activities, its workers, and the vaccine itself particularly in insecure regions such as those close to the Afghiastan-Pakistan border. For example, the house-to-house vaccination strategy is less successful in areas governed by the Taliban as some people are concerned that workers are disguised government agents and that door-markings (i.e. external evidence placed by polio workers that indicates the team has visited the house, children have been immunized, or the house needs to be revisited) are intended for drone attacks. One key informant interviewee remarked:

*Our strategy for reaching every child is house to house campaign, but we changed it because of Taliban. We changed this in the last 2 years and they don’t allow door marking and we had to change and skip door marking. Now, we do site to site or mosque to mosque campaigns and we are missing many children, but still it is better than none. We cannot wait for the permission of house to house campaigns this is also difficult, they said many times that they would allow us for house to house campaigns, then something happens and they reject it. For example, in Helmand, they said that they would allow us for house to house campaign. Suddenly, the Taliban shadow governor died in a drone attack and their decision was reversed. We still do not have permission. Likewise, in Kandahar, due to military operations the decision is withdrawn. Despite the fact the polio program is a neutral and non-political but sometimes some people make negative propaganda and say it is used for spying on Taliban and they are scared.” -* KII Afghanistan, National level

The country has also experienced substantial program fatigue among both health workers and community members. In some areas, community members demand other health services because the polio program is the only service the communities receive. One KII respondent noted:

*… it [the polio program] raises expectations that the government should do more. Sometimes the polio program is taken as hostage. People do not allow vaccination because there is no drinking water or too much garbage in a village nobody plans to pick them up. They say why are they being givent the polio vaccine while their basic needs are not addressed.* - KII Afghanistan, National level

Another respondent reflected on community fatigue and community needs:

*Now the community is tired of vaccination and they want change in the program. They request other things beside vaccination such as services I mentioned before, clean drinking water, access to other health services, therefore the interest of the people has been decreased with the program. Everything is repeated so many times and too much repetition has happened. -* KII Afghanistan, Subnational level

In addition to demanding more services, communities have historically been resistant to receiving services from male workers. In response, the program has actively recruited and employed female workers. Health workers improved their knowledge of how to engage with communities and be more responsive to their needs and this knowledge has been applied to other health programs. A key informant noted:

*Our volunteers in villages distributed bed net and nutrition materials to children who are suffering from malnutrition through this system, in southern areas for the encouragement, we give nutrition materials to those who bring their children for vaccination, after the vaccination the vaccinators give a package of nutrition materials to them, and it is effective in some areas …*” *-* KII Afghanistan, National level

This combination of skills and programmatic services has also helped to alleviate fatigue in communities that demanded additional health services.

### Case study: Nigeria

#### Respondent characteristics

Of the 953 individual who consented to participate in Nigeria’s survey, 921 completed responses were included in analysis. The 806 face to face interviews were conducted among states in the six geopolitical zones; South West (Ondo:100, Oyo:100 and Lagos:36), South-South (Bayelsa:75), South-east (Anambra:103), North West (Kano:102, Sokoto:46), North central (Nasarawa:100, FCT:117), North East (Borno:27). One hundred and fifteen individuals completed the online survey. Of the 29 KIIs conducted, 10 were involved in polio eradication at the national level, 13 were involved at the sub-national level, and 6 were frontline health workers. Table 2 outlines the survey and KII respondents, their levels of involvement in the polio program, and organizational representation.

As in the Afghanistan study, survey respondents were asked to identify barriers to program success for each polio-related activity they were engaged in (see Table 3). The most frequently reported barriers for respondents in Nigeria were related to the external setting, followed by the process of GPEI program implementation, individual barriers, organizational barriers, and lastly, GPEI program characteristics. The majority of respondents 602 (62.3%) reported experiencing external barriers The main external barriers were the economic context (27.7% of all external barriers), social factors (23.4% of all external barriers), and political factors (21.7% of all external barriers). The external context emerged as the most significant challenge for those involved in community engagement (identified as a barrier by 40% of those involved in community engagement).

#### Conflict and insecurity

Eradication efforts in Nigeria have been complicated by civil unrest and insurgent occupation which makes some areas inaccessible to health workers who fear for their safety and security. This was clearly highlighted in the key informant interviews. One interviewee in Nigeria said:

*… Well I probably think that the challenges that the polio program faces is mostly in Northern Nigeria because most of the high risk states are in Northern Nigeria. I think the major problem is in the North East particularly the states of Borno, Yobe and parts of Adamawa state and this is mainly due to the Boko-Haram insurgence, because as you know if you are unable to access a community, there is no way you can provide vaccines to the children and there is no way you can also carry out surveillance activities. And if you cannot get these indicators, then there is no way you can be sure that there is no wild polio virus circulating in certain parts*. *–* KII Nigeria, National Level

The concerns have been heightened by militants of the Boko Haram in Northern Nigeria who have actively threatened the safety of polio workers.

Survey and KII respondents described some strategies used to reduce the negative influence of conflict and insecurity on polio program activities. One approach included the coordination of polio activities with military forces to gain access to unsafe areas. Military personnel were asked to adapt to either serve as health workers (with some basic training in vaccination) or to serve as escorts to health worker cadres. The ‘Hit and Run’ strategy was also developed to increase vaccination coverage in insurgent areas in Northern Nigeria. This strategy relied on rapid and covert vaccination activities in conflict areas in order not to attract the attention of insurgents. Such immunization campaigns were conducted without any prior public announcements; health workers discreetly went into communities, vaccinated as many children as possible within a stipulated period, and then left. Despite these strategies there were low numbers for turnout and immunization in insurgent areas compared to non-insurgent areas. One national-level interview respondent further described these strategies:

*… And in communities that are partially accessible, what is done is ‘the reaching every settlement strategy’ where health workers supported by civilian Joint Task Force on a monthly basis go to these partially accessible communities and vaccinate. There is also environmental sweep sample collection, conducted in inaccessible or partially accessible communities whenever these military men or healthcare workers supported by the Joint Task Force. Whenever they access those communities they will take samples from the gutter or sewage drainage systems so that they will investigate and test them for the presence of wild polio virus. So different techniques and strategies have been developed to address issues of hard to reach areas or inaccessible communities and all that.* - KII Nigeria, National Level

#### Competing political priorities

Political leaders in these conflict-affected areas have faced competing political priorities such as balancing politically savvy messaging and other health priorities for the country. The Nigerian government has historically been supportive of the polio program and provided high-profile endorsements. However these efforts have been met with complaints about the polio program conflicting and occasionally overlapping with other health programs implemented at the state and local government levels. One KII respondent from Nigeria reflected:

*There are times whereby there are clashes of activities, there may be a polio program, side by side with another equally important program, so in that case there are lots of clashes and you know it’s always not easy. I mean in some other cases, there are so many activities while the state is planning its own, maybe the state ministry is calling you for one other activity, the national officials are planning their own. There was a scenario whereby we were having the last Outbreak Response and the officials at the national level were coming with the community health influencers promoter services program which has immunization as a component, so there is always this clash from below, within and above, so it’s not always easy.* – KII Nigeria, Subnational Level

#### Community engagement

Continuous engagement with communities in conflict areas has been an important strategy for the polio program. In Nigeria, 45.1% of the survey respondents saw the social environment as the greatest external facilitator to program success. Community engagement approaches were widely lauded in the KIIs as solutions to reaching hard-to-reach populations. One interviewee in Nigeria described such approaches:

*We identify people in a community who are respected, such as those in the womens’ groups, youth groups, retired people, or the traditional rulers. The traditional ruler is usually the patron. So, these social groups make sure that the people listen to us. Then we get them to be part of the committee that oversees immunization and other primary health care activities. Because these leaders are part of that group, it is a way to get the buy in of the community and participation when there are programs. Even though they are not paid because it is a voluntary organization, but what we do is when there are programs and we want to now recruit people and volunteers, we can say okay do you have children? give us your children, we will now involve their children and that way they are happy.* –KII Nigeria, National level

The engagement of both religious and community leaders for the mobilization of communities has played a critical role in the polio program activities. Advocacy visits were paid to these leaders to enhance their understanding of the importance of the program and the necessity of repeated immunization activities. Efforts were also made to address religious and social misconceptions about the program through community engagement. Furthermore, collaborations were fostered with community informants to close surveillance gaps through continuous community engagement and sensitization. A key-informant at the National level said:

*We use all kinds of approaches, to reach every single child. So, lots of work on community informants, so we all got 400 community informants to support inaccessible areas, people that live there to give us information* – KII Nigeria, National Level

Another KII respondent in Nigeria also reflected on the importance of female workers,

*During pre- implementation, during the selection, we encourage the ward focal person to select females so that we will not have challenges of entering house. Meanwhile the mobilizers are men because they have access to the men, the husbands, men.* – KII Nigeria, Sub-national Level

KII respondents emphasized the importance of knowing community members and the available community resources well in order to reach every child. The critical role of gaining the trust of the community members and establishing relationships with community leaders and others who are familiar with the communities, including how to locate streets and households, was highlighted. In Northern Nigeria where only female health workers are allowed to enter the houses, having a good knowledge of such communities and ensuring that vaccinators that are recruited were mostly females helped in planning of the polio programs. One interviewee in Nigeria described this in detail:

*We have improved. Right now we are developing our Reaching Every Ward micro plan which we will do for the national program too. That is what we are doing now, we are not only reaching every ward, we are expected to reach every child and it is when you are familiar with the community, the settlement, the streets, the households, that you can actually get every child involved. Our program is now based on household not on family, you know the family will involve the father, the mother and children, but the household involves the mother, mainly and her children, so in a family you could have 4 or 5 households and you could have 2 households depending on the number of wives, so that has greatly improved the reaching of every child.* – KII Nigeria, frontline health worker

#### Mistrust, Hesitancy, and Fatigue

Even though community engagement has been integral to polio eradication activities, survey respondents identified social barriers to the program as the largest external barrier for Nigeria (25% of external barriers identified). This is evidenced by the mistrust and rejection of the program activities and health workers by community members. These factors coupled with fatigue have amplified low-turnout for polio campaigns and routine immunization. One interviewee noted:

*I’m not sure whether it is external or internal fatigue. You’ve been doing this for 30 years, right? At some point, even the donors are tired, program teams are tired. it’s just the fatigue of all involved. Even the parents, the recipients are tired, they are tired of every other 2 months, people are knocking on their door with vaccines-* KII Nigeria, National Level

Similarly, healthcare workers serving these challenging contexts for many years are also increasingly fatigued and frustrated with an unresponsive health systems. A respondent reflected on organizational concerns in terms of financing and infrastructure saying:

*… funds are always not sufficient to carry out planned activities, again equipment, infrastructure are not always sufficient. And again, there are the issues of acceptability and attitude of staff. –* KII Nigeria, Sub-national level

Multiple KII respondents commented that not only did they have insufficient health care workers, but barely over half of their wards were equipped with cold chain systems.

One of the respondents also reported that the GPEI program conducted special trainings on surveillance and vaccine vial monitors, to increase health worker motivation. This has also expanded worker roles and responsibilities.

*The polio program is bringing dynamic and positive change particularly in the area of capacity, the capacity of the health care providers is all the time being developed, there is training, retraining, orientation, supportive supervision, there is accessibility. –* KII Nigeria, Sub-national level

Consequently, this combination of skills and programmatic services has also helped to alleviate fatigue in communities that demanded additional health services.

## Discussion

Significant progress has been made in reducing the burden of polio in both Afghanistan and Nigeria; however, significant challenges and opportunities remain. While the sociopolitical contexts of both countries are different, shared implementation challenges (e.g. insecurity), and strategies for addressing these barriers (e.g. community engagement) have emerged across these disparate contexts. Findings from these two countries indicate that the the main challenges hindering the eradication process of poliovirus include: 1) insecurity and armed conflicts, 2) competing political priorities and, 3) mistrust, hesitancy, and fatigue at both the community and healthworker level. While these challenges are intricately linked across the SEM levels in both countries, the scale and mechanisms through which they emerge are different.

Insecurity and armed conflict have played a particularly important role in hindering polio eradication progress in these two countries. As of June 2018, there were over 100 districts across Afghanistan where access to humanitarian actors was limited due to security risks posed by active fighting and due to constraints imposed by non-state armed groups [16]. The most recent IMB report on GPEI activities highlighted how these issues have led to one million missed children in 2018, and a lack of implementation of planned vaccine activities in June 2019 [17]. Nigeria faces similar challenges with insecurity and armed conflict, but this is more localized to Northern Nigeria and involves regional actors unlike in Afghanistan. Northern Nigeria, is the location of thousands of families who have been displaced by natural disasters and insurgency [18]. These two countries demonstrate how larger environmental barriers such as conflict can influence individuals, communities, and the political climate. While the scale of these barriers are different across the two countries, the results have been similar in that they have significantly hampered national and global goals of the GPEI. The strategies for addressing these barriers in both countries are also different. Successful strategies such as negotiating “Days of Tranquility” have been more consultative in Afghanistan while in Nigeria the use of military force and externally-driven interventions such as the “Hit and Run” approach have yielded some positive results. The difference in strategic approaches contrasts the root causes of these barriers, even though the barriers have similar manifestations in different contexts. It further highlights the importance of understanding the underlying mechanisms by which implementation barriers occur in order to appropriately target strategies.

The 16th IMB report, published in 2018 warns about a prevalent sense of fatigue and low spirit permeating the GPEI. It also shares concerns of some program leaders on whether eradication would be even possible in the next few years. The report recommends that GPEI leadership should promptly assess the effectiveness of all staff in key areas, with particular emphasis on fatigue levels, skills and experience, and dysfunctional teams to ensure these factors do not demotivate those working for the polio program [19]. This indicates an intricate connectedness of human resources, community engagement, social barriers, and ongoing conflict which is reflected in the data that emerged from both the Afghanistan and Nigerian contexts. Unlike conflict and insecurity, the prevalent sense of fatigue and low spirit permeating the GPEI in both countries may have the same root causes, which are externally-driven and linked to the four pillar strategies of the GPEI (routine immunization, supplementary immunization, surveillance, and targeted “mop-up” campaigns) and how they are implemented. Thus, overcoming the fatigue may call for fundamental changes in how the pillar strategies are implemented in different countries.

Whereas mistrust of polio program activities and workers has emerged as a major barrier in both countries, the mistrust in Afghanistan seems to have been compounded by the unintended consequences of GPEI program activities, e.g. house marking for tracking vaccination and surveillance which some have misconstrued as a possible way to mark drone targets in communities with ongoing conflicts. Hence, it is important that program activities are co-developed between program workers and community members to ensure common understanding of the strategic objectives and build trust.

While many of these challenges are ongoing in both settings community engagement strategies have been integral to improving vaccination rates and reaching hard-to-reach populations. Strategic engagement of religious and political leaders has improved community perceptions of polio program, helped correct misconceptions about polio immunization, and has built trust within communities. The engagement of community leaders has also been described as a useful intervention in other countries with larger Muslim communities [20]. These approaches have demonstrated the importance of both mutual trust and transparency between stakeholders in the planning and implementation of health programs.

These findings on both the challenges and strategies used in conflict-settings illuminate important lessons-learned for these and other similar contexts. Community needs should be holistically assessed and addressed incorporating perspectives across the community (from leadership to vulnerable groups) and the health workers who provide the services. At an individual level this could include empowering and engaging female frontline workers who often provide much-needed access to conservative households but continue to face concerns with safety and their well-being. At a systems-level, EOC structures which are widely used by these two countries have been leveraged in other disease control programs including the Ebola epidemic in West Africa [21]. Similarly, training healthcare workers in the delivery of immunization and also in surveillance of other notifiable diseases provides a more comprehensive view of a community’s health status and illuminates potential needs and services. To maintain engagement with hard-to-reach communities, disease control and eradication programs should leverage and fully support existing resources (from individuals to organizations) to meet the wider needs of these vulnerable populations.

### Strengths and limitations

This is a mixed-methods study that utilizes the strengths of both quantitative and qualitative data to robustly describe and explain the challenges, solutions, and unintended consequences of polio eradication. Both countries recruited participants at the sub-national and frontline levels, providing a breadth of knowledge and experiences. The cross-country case comparison approach also provides unique insights into conflict-affected polio endemic country experiences. While both country teams reached a large sample of their polio universe, the online survey-design posed a substantial challenge to data collection. Both teams had to innovate in their approach to reach respondents by using phone-based and in-person surveying techniques.

## Conclusion

The polio programs in Nigeria and Afghanistan have struggled to reach environmentally and socially hard-to-reach populations. Engaging stakeholders early and often, even those with extreme opposing views, is key to success. These countries’ shared lessons learned in navigating conflict and insecurity through collaborative approaches and community engagement can be brought to bear for other health programs seeking to facilitate access and achieve robust coverage.

## Data Availability

Data and publications from this project will be open access and available via an online repository.

## Abbreviations

cVDPV: Circulating vaccine derived poliovirus
CFIR: Consolidated Framework for Implementation Research
EOC: Emergency Operation Centers
EPI: Expanded Programme on Immunization
GPEI: Global Polio Eradication Initiative
IMB: Independent Monitoring Board
JHU: Johns Hopkins University
KII: Key informant interviews
NGOs: Non-government organizations
SEM: Socioecological model
SIA: Supplementary immunization activities
STRIPE: Synthesis and Translation of Research and Innovations in Polio Eradication
UNICEF: United Nations Children’s Fund
WHO: World Health Organization
WPV: Wild polio virus
